# Application value of Ki67 and serum CA125 in the deep myometrial invasion of endometrial adenocarcinoma

**DOI:** 10.1186/s12885-023-10711-x

**Published:** 2023-03-14

**Authors:** Lin Qin

**Affiliations:** Senior Department of Obstetrics & Gynecology, The Seventh Medical Center of PLA General Hospital, Beijing, China

**Keywords:** Endometrial cancer, Ki67, CA125 Antigen

## Abstract

**Objective:**

To investigate the application value of Ki67 and serum CA125 in diagnosing the deep myometrial invasion of endometrial adenocarcinoma.

**Methods:**

This study retrospectively analyzed 80 patients with endometrial adenocarcinoma, who underwent procedure from January 2018 to June 2021 at Senior Department of Obstetrics & Gynecology, the Seventh Medical Center of PLA General Hospital assigned to the Fourth Medical Center. The general clinical data, serum CA125 and Ki67 levels were compared between the superficial muscular infiltration group and the deep myometrial invasion group. We investigated the application value of Ki67 and serum CA125 in diagnosing the deep myometrial invasion of endometrial adenocarcinoma by the ROC curve.

**Results:**

80 patients were retrospectively analyzed, and 53 cases were superficial muscular infiltration, 27 cases were deep myometrial invasion. There was significant difference in age, tumor diameter, lymph node metastasis, Ki67, serum CA125, p53 status, serum CA125 and Ki67 levels between the two groups (*p* < 0.05). As high as 35% of Ki67 was the optimal cutoff value for predicting DMI in endometrial adenocarcinoma, and the area under ROC curve was 0.691, the sensitivity and specificity of diagnosis were 88.9% and 56.6%. As high as 43.645 U/ml of serum CA125 was the optimal cutoff value for predicting DMI in endometrial adenocarcinoma, and the area under ROC curve was 0.668, the sensitivity and specificity of diagnosis were 40.7% and 92.5%. After combined detection of both, the area under ROC curve was 0.719, and its sensitivity and specificity of diagnosis were 96.3% and 43.4%.

**Conclusion:**

Serum CA125 and Ki67 may be used to evaluate DMI in patients with endometrial adenocarcinoma, and the diagnostic value of combination is higher, which provide reference for clinical treatment.

## Introduction

The incidence rate of endometrial cancer (EC) has been increasing in recent years, and it has a tendency of being younger [1–3], and the 5-year survival rate is also gradually decreasing [4]. The prognosis of endometrial cancer is better than that of cervical cancer and ovarian cancer, which are two kinds of female reproductive system tumors, due to the comprehensive treatment mainly based on surgery [5]. In the staging of endometrial carcinoma, the depth of myometrial invasion is a diagnostic indicator [6], that is, the superficial myometrial invasion (invasion depth < 1/2 of the muscle layer) is stage IA, the deep myometrial invasion (DMI) is stage IB (invasion depth ≥ 1/2 of the muscle layer), and DMI is a high-risk factor for EC, with a high risk of lymph node metastasis and recurrence [7], affecting the survival time of the patients [8]. Meta-Analysis showed that DMI does not appear as an independent prognostic factor for overall survival (OS) in EC patients; instead, it seems to affect the risk of recurrence independently [9]. With the further development of medicine, we are also exploring other inspection indicators under the background that imaging is used to judge the depth of EC myometrial invasion. Ki67, a non histone protein located in the nucleus, is a value-added marker of tumor grading, which can reflect the value-added rate of malignant tumors [10]. Ki67 is an indicator of cell activity and proliferation. Its gene is located on chromosome 10, and its protein product is located in the nucleus. It starts to appear in the late stage of cell proliferation G1, gradually increases in the S and G2 stages, reaches the peak in the M stage, rapidly degrades and disappears after mitosis, and has no expression in the G0 stage. The expression of ki67 is closely related to the proliferation and differentiation of cells, and is often used as an indicator of tumor genesis, development and cell activity. Data can be obtained from the immunohistochemical results at the time of initial diagnosis. Serum carbohydrate antigen 125 (CA125) mainly exists on the surface of female genital epithelial cells, that is, it is expressed in oviduct, endometrium and ovarian cells originating from Mullerian tube. CA125 is an immuno globulin G(IgG) glycoprotein with high molecular weight, consisting of some small subunits, and its molecular weight is between 220 and 1000 kDa. DNA sequencing showed that CA125 was a transmembrane glycoprotein MUC16, containing 5797 base pairs. Based on the above theoretical basis, this study aims to explore the relationship between Ki67 and serum CA125 and the deep myometrial invasion of endometrial carcinoma. The results are reported as follows.

## Materials and methods

### The general materials

These data were retrospectively reviewed. This study was approved by ethics committees of Senior Department of Obstetrics & Gynecology of PLA General Hospital having approval no. 2022KY100-KS001 on August 25, 2022. Selected the endometrial cancer patients who were operated on in our hospital from January 2018 to June 2021 as the research objects, and screened according to the inclusion criteria. A total of 80 patients were enrolled, aged 38–83 years, with an average age of 58.79 ± 10.0 years, using the FIGO 2009 EC surgical pathology staging criteria [6].

Inclusion criteria of the study group: (1) The tumor originated from endometrium, and pathological type was endometrial adenocarcinoma; (2) Surgical treatment was performed in our hospital, and the initial treatment was surgical treatment; (3) Complete clinical and pathological data; (4) Serum CA125 was evaluated up to 1 month before the intervention.

Exclusion criteria: (1) Complicated with other system malignancies; (2) Complicated with serious medical diseases, which affects the scope of surgery; (3) Radiochemotherapy or endocrine therapy before operation. Strictly follow the inclusion and exclusion criteria, and select inpatients in the same period to control selection bias.

Among the 80 patients studied in this group, 53 patients were in stage I (66.25%, 53/80), 11 patients in stage II (13.75%, 11/80), 15 patients in stage III (18.75%, 15/80), and 1 patient in stage IV (1.25%, 1/80). They were divided into two groups: 53 cases (53/80, 66.2%) were the superficial muscular infiltration group (muscle layer infiltration depth < 1/2); 27 cases (27/80, 33.7%) were the deep myometrial invasion group (muscle infiltration depth ≥ 1/2).

### Clinical data collection

The electronic cases of all enrolled patients were checked from the medical record system of our hospital, and the following information was extracted from the medical records, inspection reports and pathological reports: age, body mass index (BMI), tumor diameter, pathological stage, lymph node metastasis status, Ki67 value, serum CA125 value and P53 status.

Load the CA125 electrochemiluminescence kit (Roche Diagnostics GmbH,Shanghai, Germany, Cat. No. 2157-10) onto the Cobas e801 fully automatic series electrochemiluminescence instrument equipment, and use the chemiluminescence method to detect the serum CA125 value. The normal range value is 0-35U/ml. Ki67 was detected by immunohistochemistry in the same laboratory with Ki67 antibody reagent (clone number MXR002, Fuzhou Meixin Biotechnology Development Co., Ltd., Fujian, China), using the LEICA BOND MAX automatic immunohistochemical staining system.

### Statistical analysis

SPSS 23.0 software was used for statistical analysis, and the counting data were compared by Chi-square test or Fisher exact test. Compliance with normal distribution was verified with the Shapiro-Wilk test. For the measurement data that do not conform to the normal distribution, the median and quartile were used to express the data. The Mann-Whitney U-test was used to compare the two groups. The measurement data subject to normal distribution were expressed by mean ± standard deviation, and the differences were assessed using Student’s t-test. Using the receiver operating characteristic (ROC) curve, analyze the predictive value of Ki67 and serum CA125 levels on deep myometrial invasion in EC patients, and determine the best cut-off value according to the Youden Index.

## Results

### Comparison of general data between two groups

There were significant differences in age, tumor diameter, lymph node metastasis and P53 status between the two groups (*p*<0.05), while there was no significant difference in BMI between the two groups(*p*>0.05). Shown as Table 1.

**Table 1:** Comparison of general data between two groups

Groups	n	age(years)	BMI	tumor diameter(cm)	LNM(%)	P53 positive(%)
superficial myometrial invasion	53	57.02 ± 8.982	26.21 ± 3.645	2.284 ± 1.232	3.77(2/53)	30.19(16/53)
DMI	27	62.26 ± 11.096	26.96 ± 3.787	2.948 ± 1.290	29.63(8/27)	59.26(16/27)
χ^2^/t value		-2.276	-0.865	-2.244	10.933	6.299
*P* value		0.026	0.390	0.028	0.002	0.016

### Comparison of serum CA125 and Ki67 levels between two groups

There was a statistically significant difference in serum CA125 and Ki67 levels between the two groups(*p*<0.05). Shown as Table 2.

**Table 2:** Comparison of serum CA125 and Ki67 levels between two groups

Groups	n	CA125	Ki67
superficial myometrial invasion	53	17(10.63–25.75)	41.08 ± 24.73
DMI	27	28.54(13.8–65.8)	56.85 ± 17.60
t/Z value		-2.447	-3.288
*p* value		0.014	0.002

### ROC curve analysis of Ki67 and serum CA125 in predicting DMI with EC patients

**Fig. 1:**
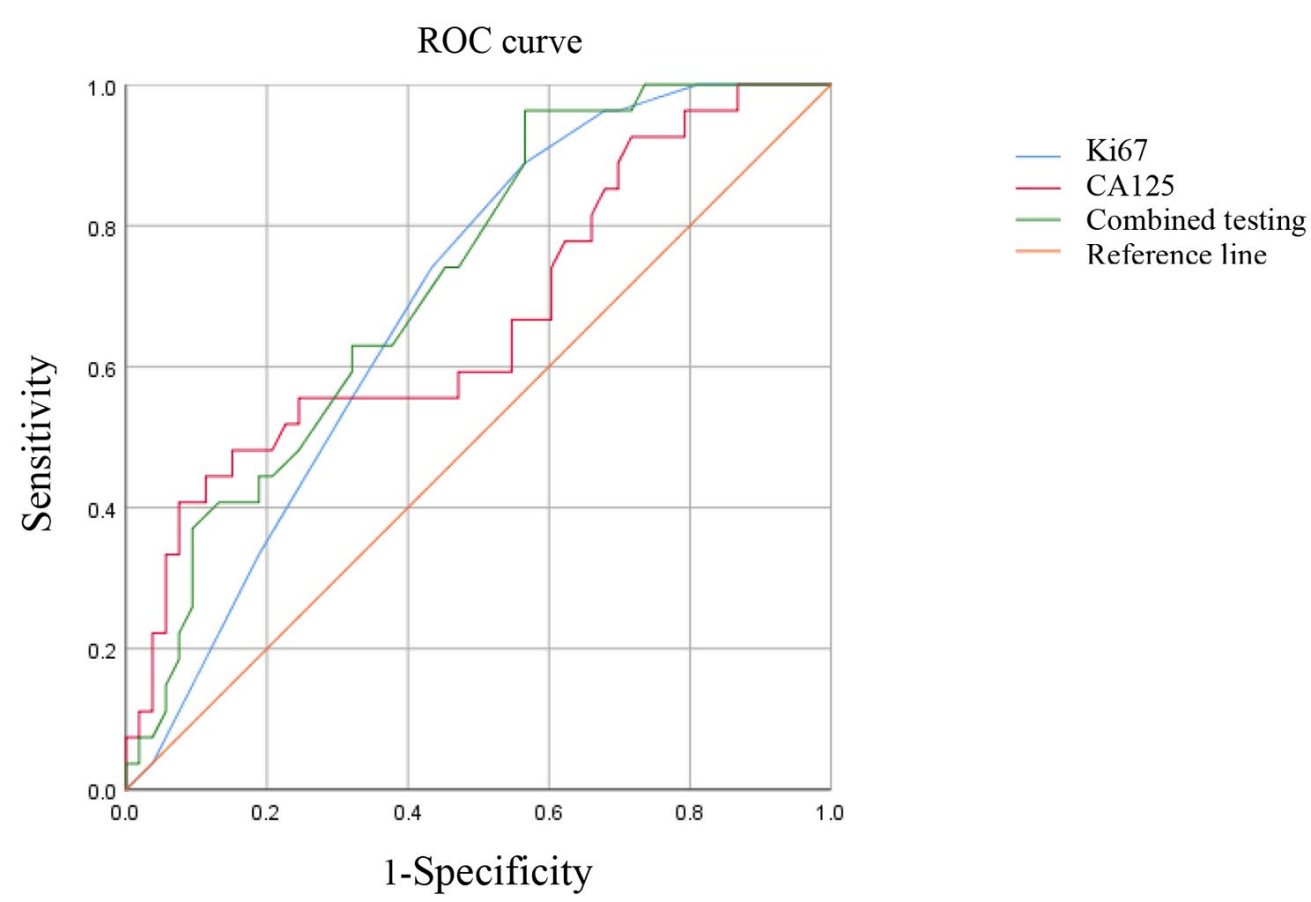
ROC curve analysis of Ki67, serum CA125 and combined both in predicting DMI with EC patients

Ki67, serum CA125 and their combined detection have diagnostic efficacy for DMI in EC patients. The area under the curve (AUC) of the combined diagnosis of Ki67 and serum CA125 is 0.719, which has the highest diagnostic efficacy. Shown as Fig. 1; Table 3.

**Table 3:** The value of Ki67 and serum CA125 detection alone and combined both in predicting DMI with EC patients

Index	Critical value	Youden Index	AUC	95%*CI*	Sensitivity(%)	Specificity(%)	*p* value
Ki67(%)	35	0.323	0.691	0.577–0.805	88.9%	56.6%	0.005
CA125(U/ml)	43.645	0.332	0.668	0.538–0.798	40.7%	92.5%	0.014
Combined both		0.397	0.719	0.606–0.831	96.3%	43.4%	0.001

## Discussion

The incidence rate of endometrial cancer has increased year by year in recent years, ranking the first among reproductive system tumors in developed countries [11]. This research only studied endometrioid adenocarcinoma, and uterine sarcoma [12] was not included in the study. The main indicators of the “Mayo Standard” include that the tumor invades less than 1/2 of the myometrium. In 2021, the European Society of Gynaecological Oncology (ESGO) will define the population with four characteristics of stage I endometrial carcinoma, histologic grade G1-G2, myometrial invasion < 50%, and no lymphatic vascular space invasion as low-risk population, and such patients do not need further adjuvant treatment after surgery [13]. Lakhwani et al. [14] showed that the depth of muscular invasion and histological grading were two important prognostic factors in EC. Other studies had shown that the prognosis of EC was related to lymph node metastasis and muscular invasion [15, 16]. Therefore, this study conducted a study on the depth of myometrial invasion in order to play a certain role in the treatment, prognosis and follow-up of endometrial cancer. Imaging technology is often used in clinical diagnosis of EC myometrial invasion [17, 18]. Research showed that the sensitivity and specificity of MRI in diagnosing uterine myometrial invasion were 70% and 92% respectively [19]. The sensitivity and specificity of Doppler ultrasound in diagnosing myometrial invasion were 67% and 81.7% respectively [20]. Ambrosio et al. [21] showed that preoperative ultrasound tumor size does not appear as a prognostic factor in EC women. Frühauf et al. [22] showed that myometrial invasion in EC was evaluated by subjective assessment using ultrasound (< 50% or ≥ 50%) and calculated as tumor/uterine anteroposterior diameter ratio (Karlsson’s ratio), which had 56.3% sensitivity, 76.4% specificity, and 68.1% overall accuracy. A combination model may improve the detection rate of DMI before surgery and have an extraordinary impact on EC management in the modern view of a patient-tailored approach. Imaging is more expensive than blood drawing examination, and some patients cannot perform MRI examination due to the placement of stents, steel plates, intrauterine devices and other reasons, so this study explored the diagnostic value of Ki67 and serum CA125 in the deep muscle layer invasion of endometrial cancer. Before treatment, the serum CA125 value can be obtained by blood sampling. The initial diagnosis of endometrial cancer patients is mostly obtained by curettage pathology, and Ki67 value can be obtained by immunohistochemistry (IHC) examination at the same time.

P53 protein can inhibit tumor occurrence and cell apoptosis. It is an important tumor suppressor gene. It can induce tumor cell apoptosis through multiple signal transduction pathways. If point mutation occurs, it will lose its anti-tumor effect and cause malignant transformation of cells [23]. Arend et al. [24] showed that 10 − 20% of EC and 90% of serous carcinoma can have P53 mutation. Talhouk et al. [25] showed that P53 was mostly expressed in high-grade, poor prognosis non endometrioid EC. The results of this study are consistent with the above results, suggesting that P53 mutant is a related factor affecting the deep myometrial invasion of EC.

Ki67 antigen is a nuclear proliferation marker gene, which mainly reflects the proliferation status of cells. At present, it is widely used in clinical as a proliferation marker of tumor cells, and its expression level can provide effective disease information for clinicians [26, 27]. It is located on chromosome 10 and is a protein encoded by MKi-67 gene. Its content increases significantly in the active phase of cell division, that is, its expression gradually increases in G1, S, G2 and M phases of cell division cycle, but it does not express in G0 phase of cell quiescence. Therefore, in the pathological report, the index is closely related to the degree of differentiation, invasion, metastasis and prognosis of many tumors. The expression of Ki67 in EC is related to prognosis [28]. This study shows that when Ki67 is higher than 35%, it is necessary to be alert to the possibility of EC deep myometrial invasion.

Serum CA125 is a cell surface antigen derived from human coelomic metaplasia. When cells derived from the reproductive epithelium of Mullerian duct show abnormal proliferation, serum C125 is significantly increased [29], which plays an important role in the diagnosis and prognosis evaluation of EC. Bian et al. [30] showed that the serum CA125 level of EC patients was significantly higher than that of normal healthy people. Another study shows that serum CA125 alone is not good for diagnosing and predicting the prognosis of EC [31]. Some studies show that CA125 combined with HE4 can diagnose early endometrial cancer, with sensitivity of 57 − 76% and specificity of 90 − 100% [32]. This study shows that when the serum CA125 is greater than 43.654U/ml, the sensitivity is not high, only 40.7%, but the specificity is as high as 92.5%. In addition, the combination of CA125 and Ki67 is more effective in the diagnosis of EC deep myometrial invasion, and the sensitivity can be as high as 96.3%. Nithin et al. [33] showed that the CA125 value increased significantly in cases of deep myometrial invasion and extrauterine metastasis, and increased with the increase of FIGO stage of EC, which is of great value in judging the progress of endometrial cancer and evaluating the prognosis.

This research represents a valid contribution to the field, since it proposes noninvasive, quick and cheap items with worth to be further studied in this application. But there are also potential limitations to our work. First, the small sample size is a shortcoming that cannot be ignored. Second, selection bias was inevitable since we only included patients with the primary endometrial adenocarcinomas. Last but not least, there was a lack of data based on gene expression, and such data may provide a clearer picture of disease heterogeneity and warrant exploration in the future.

## Conclusion

To sum up, Ki67 combined with serum CA125 has predictive value for deep myometrial invasion in patients with endometrial adenocarcinoma, and the combined detection of Ki67 and CA125 is helpful to improve the diagnostic efficiency, which is worthy of clinical promotion. However, this study still needs to be further confirmed by multi center and large sample size.

## Data Availability

The datasets generated and/or analysed during the current study are not publicly available due personal privacy but are available from the corresponding author on reasonable request.
